# Nuclear delivery of recombinant OCT4 by chitosan nanoparticles for transgene-free generation of protein-induced pluripotent stem cells

**DOI:** 10.18632/oncotarget.9276

**Published:** 2016-05-10

**Authors:** Salma Tammam, Peter Malak, Daphne Correa, Oliver Rothfuss, Azzazy Hassan M.E., Alf Lamprecht, Klaus Schulze-Osthoff

**Affiliations:** ^1^ Laboratory of Pharmaceutical Technology and Biopharmaceutics, University of Bonn, 53121 Bonn, Germany; ^2^ Department of Chemistry, The American University in Cairo, 11835 Cairo, Egypt; ^3^ Interfaculty Institute for Biochemistry, University of Tuebingen, 72076 Tuebingen, Germany; ^4^ Laboratory of Pharmaceutical Engineering, University of Franche-Comté, Besançon 25000, France; ^5^ German Cancer Consortium (DKTK) and German Cancer Research Center, 69120 Heidelberg, Germany

**Keywords:** induced pluripotent stem cells, OCT4, transgene-free stem cells, chitosan nanoparticles, reprogramming

## Abstract

Protein-based reprogramming of somatic cells is a non-genetic approach for the generation of induced pluripotent stem cells (iPSCs), whereby reprogramming factors, such as OCT4, SOX2, KLF4 and c-MYC, are delivered as functional proteins. The technique is considered safer than transgenic methods, but, unfortunately, most protein-based protocols provide very low reprogramming efficiencies. In this study, we developed exemplarily a nanoparticle (NP)-based delivery system for the reprogramming factor OCT4. To this end, we expressed human OCT4 in Sf9 insect cells using a baculoviral expression system. Recombinant OCT4 showed nuclear localization in Sf9 cells indicating proper protein folding. In comparison to soluble OCT4 protein, encapsulation of OCT4 in nuclear-targeted chitosan NPs strongly stabilized its DNA-binding activity even under cell culture conditions. OCT4-loaded NPs enabled cell treatment with high micromolar concentrations of OCT4 and successfully delivered active OCT4 into human fibroblasts. Chitosan NPs therefore provide a promising tool for the generation of transgene-free iPSCs.

## INTRODUCTION

The generation of human induced pluripotent stem cells (iPSCs) from somatic cells represents a major advancement in stem cell biology, especially because of their many potential applications including patient-specific tissue replacement, drug screening and disease modeling [1, 2]. Human somatic cells can be reprogrammed to iPSCs by the ectopic expression of the four pluripotency-related transcription factors OCT4, SOX2, KLF4 and c-MYC, also known as the OSKM factors [3, 4].

In most cases the OSKM factors are introduced via retroviral transduction, which however bears the risk of insertional mutagenesis of the genome-integrating viruses [5, 6]. Indeed, retroviral vector DNAs can insert at a large number of sites in the host genome and promote the expression of oncogenes or disrupt tumor suppressor genes. Several protocols have been explored to circumvent the integration of foreign DNA into the genome. These include the transient expression of reprogramming factors using non-integrating adenoviruses, plasmids, RNA, episomal vectors or the excision of the transgenes after reprogramming by site-specific recombinases or transposases [7–13]. While such approaches reduce the risk of insertional mutagenesis, nucleic acid-free approaches by the direct delivery of reprogramming proteins represent presumably the safest methods with respect to future clinical applications of iPSCs. In addition, the use of proteins also avoids the sustained expression of transgenes after reprogramming. Since pluripotency factors are not only crucial for stemness but also involved in tumorigenesis, their expression should be restricted to the reprogramming process [14–18].

A major hurdle for the intracellular delivery of proteins is their limited ability to cross the cell membrane. Small protein transduction domains (PTDs) from proteins (e.g., HIV-TAT) can be fused to proteins of interest to facilitate their delivery into host cells [19–21]. Nevertheless, reprogramming by PTD-bearing proteins is very slow (≈8 weeks) and inefficient (≈0.001% reprogramming rate) compared to retrovirus-based protocols (≈0.01% of input cells). The low success of protein-induced stem cell generation is presumably caused by the low stability and solubility of recombinant reprogramming factors as well as their poor endosomal release. Therefore, further developments in protein delivery systems are required to enhance the efficiency of reprogramming to iPSCs.

Polymeric nanoparticles (NPs), such as chitosan NPs, offer a more promising approach. Apart from their biocompatibility and biodegradability, their ability to encapsulate therapeutic biomolecules protects them from premature degradation [22–26]. Chitosan [poly(N-acetyl glucosamine)] is a biodegradable polysaccharide that can be used to formulate NPs by several methods. The ionotropic gelation method, which allows the incorporation of the therapeutic protein into the NPs, occurs via mild electrostatic interactions in aqueous, physiological conditions [22]. As cationic polymers chitosan NPs adhere to the negatively charged cell surface, facilitating their cellular uptake by endocytosis [27]. The presence of primary amine groups on the NP surface facilitates endosomal escape via the proton sponge effect. Moreover, tagging of nuclear localization sequences (NLS) to the chitosan NPs allows directing NPs to the cell nucleus [28].

Among the OSKM factors, the transcription factor OCT4 (POU5F1) is of particular importance for reprogramming and the self-renewal of stem cells [29]. OCT4 is highly expressed in pluripotent cells and becomes silenced upon differentiation. The precise expression level of OCT4 determines the fate of embryonic stem cells [30]. DNA binding of OCT4 to promoter regions initiates transcription of various genes involved in pluripotency or self-renewal, such as *NANOG* and *SOX2* [31–32]. OCT4 is therefore considered a master regulator for the maintenance of pluripotent cells and successful reprogramming with *OCT4* alone has been shown [33]. It was however reported that recombinant OCT4 protein has a limited solubility and stability under cell culture conditions. Furthermore, recombinant cell-permeant OCT4-TAT fusion proteins show a weak endosomal release after cellular uptake, which, in addition to their poor stability, represents another bottleneck for achieving robust reprogramming by protein transduction [34, 35].

Various expression systems are available for recombinant protein production. Several groups reported the expression of OCT4 in *E. coli* or mammalian cells [19–21, 36]. Bacterially expressed OCT4 is usually found in inclusion bodies and needs to be denatured and refolded *in vitro*. The refolding process is cumbersome and results in very poor yields of properly folded active proteins [37]. On the other hand, OCT4 expressed in mammalian cells can be purified in a native state, but production of large quantities of purified and active protein remains challenging. This limitation might be overcome by the baculoviral expression system in Sf9 insect cells, which can provide high yields of functional proteins [38].

In this study, we report the formulation of nuclear-targeted chitosan NPs for human OCT4. OCT4 was expressed in Sf9 insect cells, evaluated with respect to its activity and nuclear localization and encapsulated in chitosan NPs. Chitosan NPs were able to considerably stabilize the DNA-binding activity of recombinant OCT4 as well as to deliver the OCT4 cargo into nuclei of human fibroblasts. Our study therefore demonstrates a proof-of-concept for a DNA-free protein transduction system, making chitosan NPs a promising and safe tool for cellular reprogramming and derivation of transgene-free iPSCs.

## RESULTS

### Chitosan NP formulation and characterization

**Figure 1:**
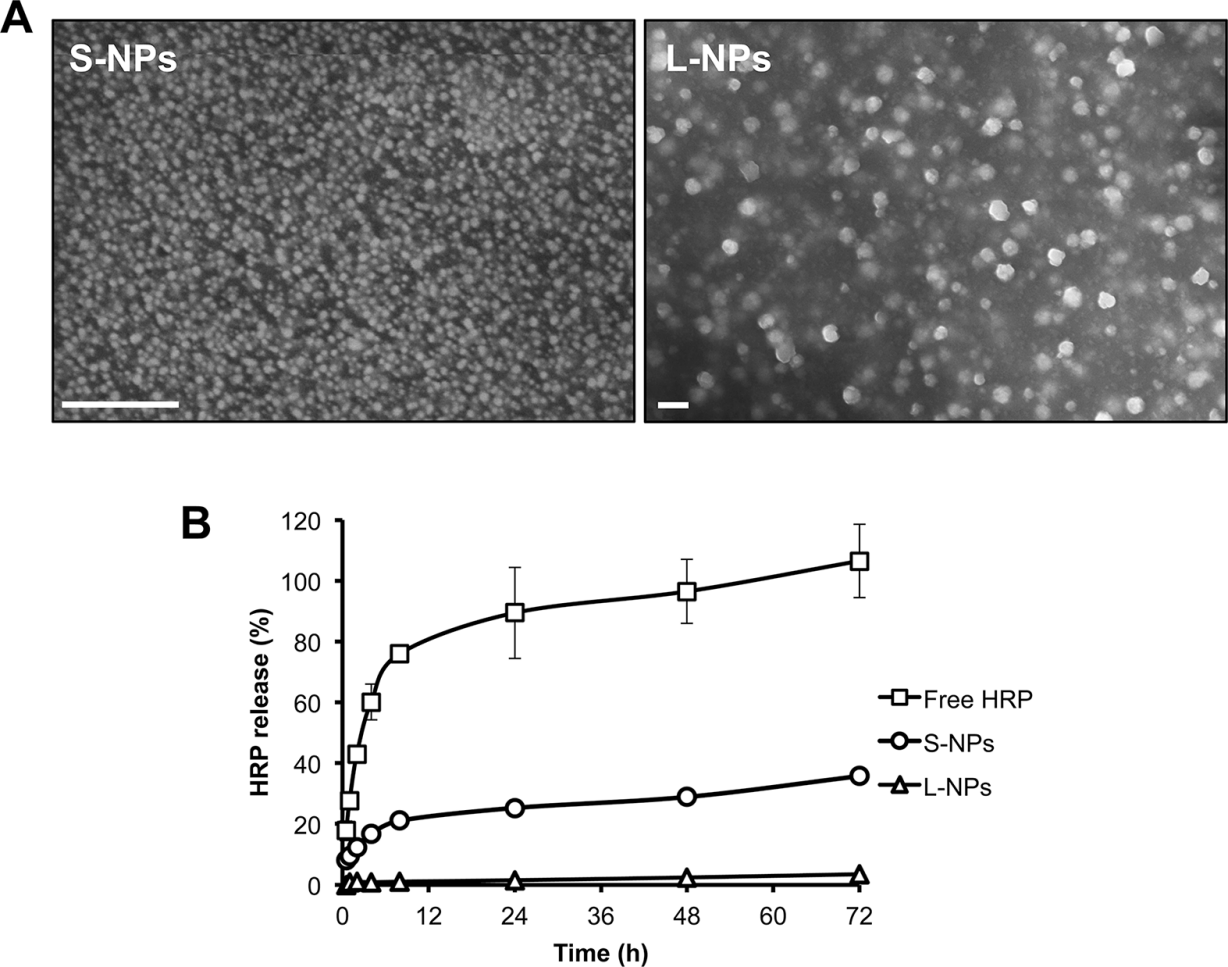
Characterization of chitosan NPs (**A**) Scanning electron micrographs from S-NPs (left) and L-NPs (right). Bars = 200 nm. (**B**) Kinetics of HRP release from S-NPs and L-NPs in comparison to free HRP, as measured by enzyme activity. Results are given as mean ± SD from three experiments performed in triplicate. Similar release profiles were obtained by measuring protein content.

To demonstrate that chitosan NPs preserve protein activity we initially encapsulated horseradish peroxidase (HRP) as a model protein and investigated the encapsulation efficiency and release profile from small (S-NPs) and large (L-NPs) nanoparticles. Scanning electron microcopy revealed that the chitosan NPs were spherical with no apparent aggregation (Figure 1A). The average hydrodynamic diameter of S-NPs and L-NPs was 25 nm and 150 nm, respectively. Both NPs had a positive zeta potential, a surface parameter affecting the stability of dispersed NPs and their cellular adsorption (Table 1). The encapsulation of HRP did not significantly change the hydrodynamic diameter of the NPs. Neither did it affect HRP activity, since encapsulation efficiency determined by either protein content or enzyme activity did not significantly differ (Table 1). Moreover, NPs of both sizes were able to release active HRP (Figure 1B). S-NP showed an initial burst during the first 2 h when ≈12% of the loaded HRP activity was released. HRP release then slowed down reaching ≈35% release within 72 h. The release of HRP from L-NPs was considerably weaker, and only ≈3.5% was released after 72 h (Figure 1B).

**Table 1:** Characterization of chitosan S-NPs and L-NPs

NP	Cargo	HD (nm)	ZP (mV)	EE % (% Activity)	EE (% Protein)
S-NP	w/o	25 ± 2	35 ± 2	-,-	-,-
	HRP	26 ± 2	22 ± 2	56.2 ± 0.0	58.7 ± 2.9
L-NP	w/o	147 ± 3	50 ± 5	-,-	-,-
	HRP	143 ± 1	45 ± 2	97.6 ± 0.4	94.3 ± 3.4

Abbreviations: EE: encapsulation efficiency; HD: hydrodynamic diameter; HRP: horseradish peroxidase; ZP: zeta potential.

### Baculoviral OCT4 expression and purification

**Figure 2:**
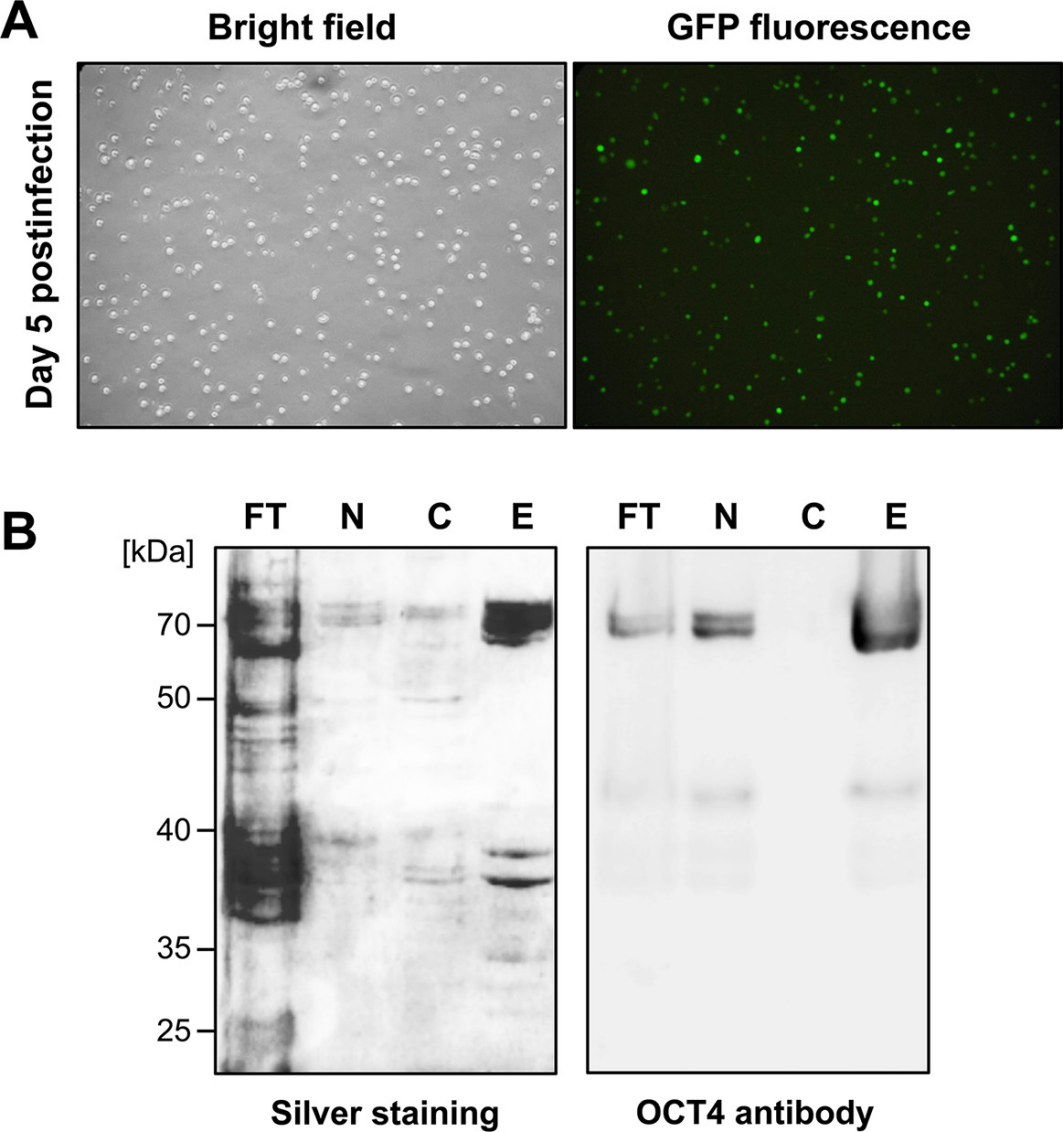
OCT4 expression and purification from Sf9 cells (**A**) Efficient Sf9 cell infection with recombinant OCT4-encoding baculoviruses was monitored by expression of GFP five days post-infection. (**B**) Recombinant OCT4 is enriched in nuclear fractions of Sf9 cells and can be purified by GST affinity chromatography, as shown by silver staining (left) and immunoblot analysis using an OCT4 antibody (right). FT: column flow-through, N: nuclear fraction, C: cytosolic fraction, E: column eluates.

For encapsulation in NP, the pluripotency factor OCT4 was expressed as a glutathione-S-transferase (GST) fusion protein in Sf9 insect cells using the baculoviral expression system. Following homologous recombination of the *GST-OCT4* cDNA with linearized wildtype baculovirus DNA, high-titer virus stocks were produced. Infection of Sf9 cells with the recombinant viruses resulted in high infection efficiencies, as monitored by expression of the *GFP* gene on the baculoviral DNA (Figure 2A). Since recombinant OCT4 was mainly localized in the nucleus of Sf9 cells (Figure 2B), we first isolated the nuclei of Sf9 cells five days post-infection. After lysis of the nuclei GST-affinity chromatography was performed. As revealed by silver staining and immunoblotting (Figure 2B), OCT4 protein could be easily enriched by this protocol, yielding ≈6 mg/l of purified OCT4 from Sf9 cell suspension cultures.

### Chitosan S-NPs stabilize OCT4 DNA-binding activity

**Figure 3:**
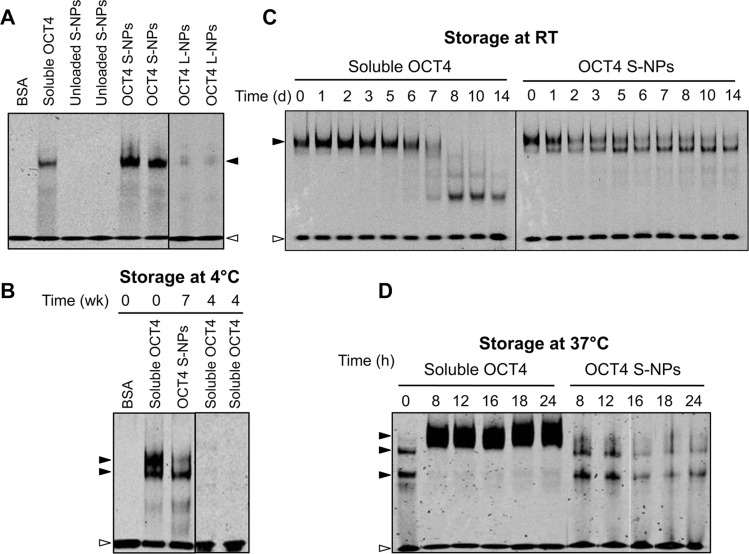
S-NP encapsulation stabilizes OCT4 DNA-binding activity (**A**) EMSA analysis showing OCT4 stabilization by S-NPs but not L-NPs. Unloaded S-NPs, soluble OCT4 protein as well as OCT4 encapsulated in S-NPs and L-NPs were subjected to EMSA analysis. The DNA-binding activity of OCT4 was analyzed using an oligonucleotide containing the OCT4 consensus motif. BSA was used as a negative control. (**B**–**D**) OCT4-loaded S-NPs stabilize OCT4 DNA-binding activity for 7 weeks at 4°C (B), for 14 days at RT (C), and for 24 h under cell culture conditions at 37°C in the presence of serum (D), whereas the DNA-binding activity of soluble OCT4 is rapidly lost under these conditions. The slowly migrating protein/DNA complex of soluble OCT4 shown in (D) is presumably caused by aggregation of the OCT4 protein under cell culture conditions. The protein amount used per lane for the EMSAs corresponds to 30 ng (A), 100 ng (B) and 250 ng (C, D). The OCT4/DNA complexes and unbound oligonucleotide are marked by closed and open arrowheads, respectively.

Recombinant OCT4 has been shown to become rapidly degraded under cell culture conditions [34]. We therefore tested the OCT4 DNA-binding activity by electrophoretic mobility shift assays using an oligonucleotide with the octamer-binding site from the Ig heavy chain enhancer. Soluble OCT4 as well as OCT4 encapsulated in S-NPs induced the appearance of a specific DNA/protein complex, which was not detectable with bovine serum albumin (BSA) as the negative control (Figure 3A) or in the presence of a 50-fold excess of unlabeled oligonucleotide (data not shown). In comparison to S-NPs, OCT4-loaded L-NPs induced a much weaker electrophoretic shift (Figure 3A). Similar results were obtained with higher L-NP concentrations (data not shown), indicating a less efficient release of OCT4 from L-NPs. Further experiments were therefore only conducted with OCT4-loaded S-NPs.

We next tested several storage conditions of the NPs for OCT4 DNA-binding. Whereas the long-term storage of OCT4-loaded NPs at 4°C still retained DNA-binding activity even after 7 weeks, no DNA-binding activity could be retained with soluble OCT4 protein (Figure 3B). Furthermore, at room temperature (RT) DNA binding of soluble OCT4 was lost within 7 days, whereas OCT4-loaded NPs showed still DNA binding after 14 days (Figure 3C). Importantly, S-NPs were able to maintain OCT4 DNA-binding activity even under cell culture conditions at 37°C (Figure 3D). In contrast, at 37°C soluble OCT4 caused the appearance of a high-molecular weight complex with reduced mobility (Figure 3D), which was presumably due to the reported precipitation and aggregation of OCT4 under cell culture conditions in the presence of serum [34, 35]. Thus, encapsulation of OCT4 in S-NPs results in a considerable stabilization of OCT4 DNA-binding activity.

### Effects of NLS density on S-NP cell binding, uptake and nuclear delivery

**Figure 4:**
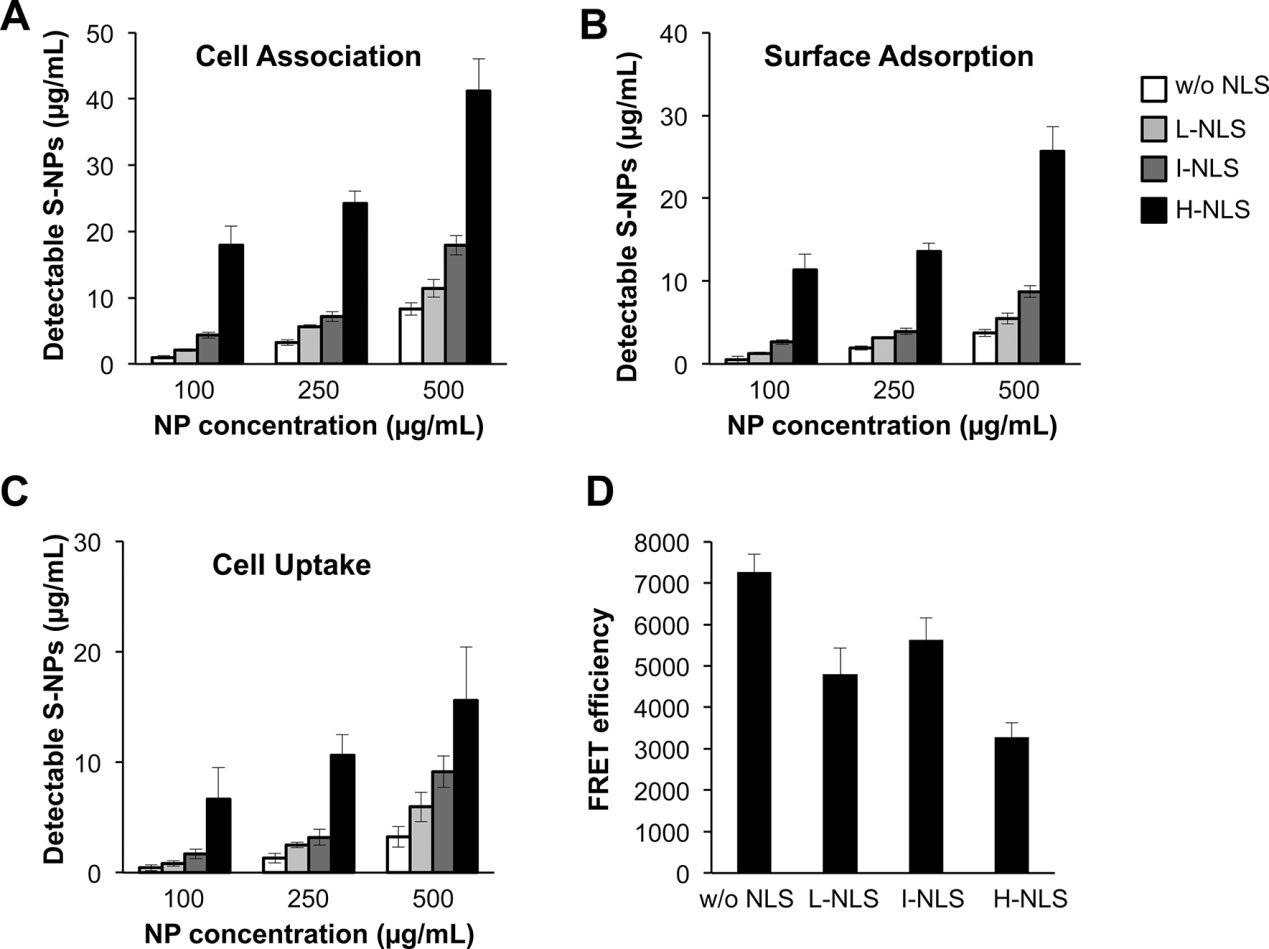
Effects of NLS density on S-NP cell surface binding, uptake and nuclear delivery (**A**–**C**) Non-modified S-NPs or S-NPs tagged with low (L), intermediate (I) or high (H) NLS densities were incubated at the indicated concentrations with human fibroblasts. After 24 h chitosan NPs were stained as detailed in Material and Methods. The recovered amount of (A) cell-associated (i.e. surface-bound and internalized) NPs, (B) NPs bound to cell surface or (C) NPs taken up intracellularly was calculated from a standard curve by fluorometry. (**D**) Effects of NLS density on S-NP nuclear delivery as assessed by FRET fluoroscopy. Human fibroblasts were treated for 24 h with 250 μg/mL of the indicated versions of S-NPs. Measurement of FRET efficiency indicates the strongest colocalization of the nuclear DNA dye with SN-Ps lacking an NLS. Results are given as means ± SD.

We next investigated whether tagging with a nuclear localization sequence (NLS) could alter the cellular uptake and nuclear delivery of S-NPs. To this end, S-NPs with different NLS densities were generated and administered at different concentrations to human dermal fibroblasts. Subsequently, NPs were labeled with FITC-coupled wheat germ agglutinin (WGA) exhibiting a high affinity to chitosan. WGA labeling was performed in permeabilized and non-permeabilized cells at different temperatures to allow the discrimination of cell-associated and internalized NPs. We found that increasing NP concentrations resulted in an elevated cell association of the NPs (Figure 4A) as well as an increased cell surface binding (Figure 4B) and cellular uptake (Figure 4C). The presence of an NLS dose-dependently increased the amount of cell surface-bound and internalized S-NPs.

FRET spectroscopy with the nuclear DNA dye Hoechst and FITC was then performed to quantify the nuclear delivery of S-NPs in human fibroblasts. Surprisingly and in contrast to the previous experiments, unmodified S-NPs revealed a higher FRET efficiency and hence an increased nuclear localization compared to NLS-modified NPs (Figure 4D). Thus, even though NLS-tagging of S-NPs increased their cellular uptake, nuclear delivery was impaired. Since OCT4 exerts its cellular function in the nucleus, further tests were conducted with unmodified S-NPs, revealing the highest nuclear delivery.

### Intracellular OCT4 delivery by S-NPs

**Figure 5:**
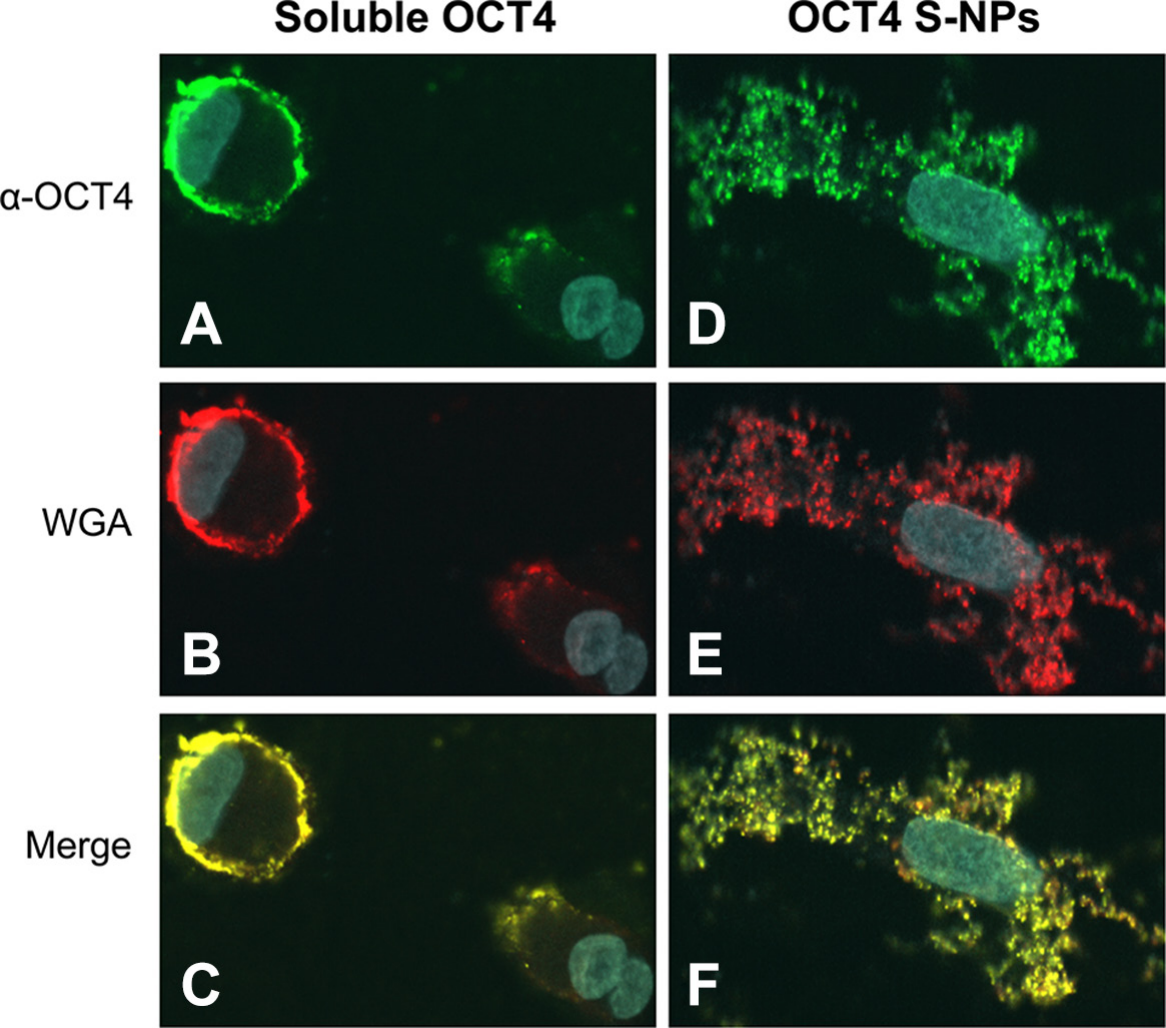
OCT4-loaded S-NPs but not soluble OCT4 protein are imported into cells and partially localize in the cell nucleus Human primary fibroblasts were treated with 50 μg of each recombinant soluble OCT4 (**A**–**C**) or OCT4 encapsulated in S-NPs (**D**–**F**). After 24 h cells were stained with OCT4 antibodies (green) or for chitosan NPs using WGA-Alexafluor 488 (red). Nuclear DNA was stained with Hoechst 33258 (blue). Merged images demonstrate that exogenous soluble OCT4 is excluded from cells and adheres to the cell membrane. In contrast, OCT4-loaded S-NPs are localized intracellularly, showing a partial overlap with Hoechst nuclear staining.

**Figure 6:**
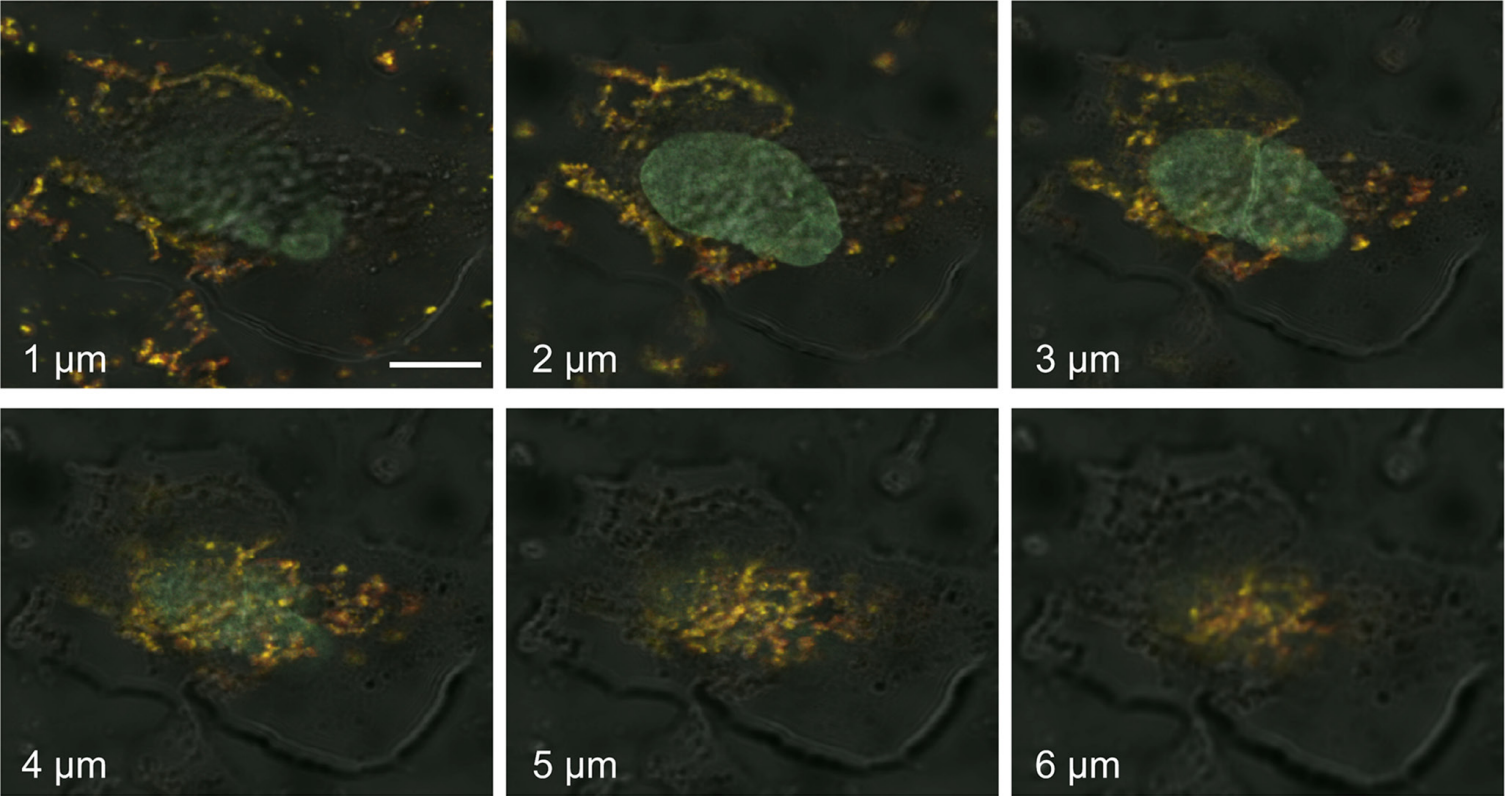
Z-stack imaging series of human fibroblasts treated with OCT4-loaded S-NPs Cells were treated for 24 h with 50 μg of the SN-Ps and then stained with OCT4 antibody (green), WGA-Alexafluor 488 (red) and Hoechst 33258 (blue). Z-stack images through the cell nucleus were collected at 1-μm steps by confocal laser scanning microscopy. The yellow fluorescence of the merged images indicates the colocalization of OCT4 and S-NPs in perinuclear and nuclear regions. The depth in micrometers at which images were taken is indicated. Scale bar = 10 μM.

We next investigated the cellular distribution of OCT4-loaded S-NPs by confocal laser scanning microscopy. To this end, human fibroblasts were treated with equal protein amounts of either soluble OCT4 or OCT4-loaded S-NPs. Soluble OCT4 was exclusively found at the cell membrane but was unable to enter the cells (Figure 5A–5C). In contrast, OCT4 encapsulated in NPs was efficiently imported into the fibroblasts, as revealed by costaining for OCT4 and chitosan using an OCT4 antibody and WGA-Alexafluor 488, respectively (Figure 5D–5E). Moreover, this intracellular distribution partially overlapped with nuclear Hoechst staining, indicating nuclear delivery of OCT4 (Figure 5F). To further analyze a nuclear delivery of the OCT4-loaded NPs in different layers of cell nucleus, confocal *Z*-stack images were collected at 1-μm steps (Figure 6). Visualization of a Z-stack indeed indicated that OCT4 encapsulated in NPs was detectable in both perinuclear and intranuclear regions of the fibroblasts.

## DISCUSSION

Various strategies have been suggested to accomplish transgene-free derivation of iPSCs, including the use of non-integrating viruses, site-specific recombinases for transgene excision, plasmids or RNA transfection [7–13]. Although these methods significantly reduce the risk of genome alterations, a nucleic acid-free system is generally preferred. Chitosan NPs are increasingly used as protein delivery vehicles due to their mild formulation conditions [22–24]. To demonstrate that chitosan NPs preserve protein activity we initially encapsulated HRP as a model protein and investigated the encapsulation efficiency and release profile. Both S-NPs and L-NPs released HRP in its active form. Since S-NPs revealed a more efficient release and better preservation of OCT4 DNA-binding, further experiments were solely conducted with S-NPs.

Compared to L-NPs, S-NPs showed a higher sustained release of active HRP over a 72-h incubation period and were able to increase OCT4 stability at 4°C (up to 7 weeks), at room temperature (up to two weeks) and more importantly in cell culture at 37°C. Under cell culture conditions the DNA-binding activity of soluble OCT4 was lost within 1 h, whereas NP-encapsulated OCT4 preserved activity throughout the 24-h period tested. Also Bonsali *et al.* reported that a rapid loss of TAT-OCT4 activity under cell culture conditions [34]. The presence of serum stabilized the TAT fusion protein but at the same time reduced its cellular uptake [35]. Moreover, while a combination of serum and serum replacement containing lipid-rich BSA improved the stability and cellular uptake of TAT-OCT4, serum replacement was cytotoxic to fibroblasts [35, 39].

An ideal protein transduction method has to fulfill several criteria and (1) stabilize the recombinant protein, (2) facilitate its cellular entry and endosomal escape, (3) allow cell treatment with sufficient protein concentrations and (4) provide sustained protein levels without the need for repeated treatments. We found that chitosan NPs were able to considerably stabilize OCT4 in cell culture conditions. The ability of chitosan NPs to associate with cells has been repeatedly reported [25, 40], however, only a few studies distinguished between cell surface-bound and internalized NPs [41–43]. Since soluble OCT4 is incapable of cell entry, we validated that chitosan NPs are capable of cellular entry rather than adhering to the cell surface. To this end, we employed a method that exploits the preferential affinity of WGA to chitosan and thereby allows the quantification of cell surface-bound and internalized chitosan NPs. In non-permeabilized cells at 4°C WGA-FITC was not internalized and labeled only extracellular chitosan NPs, whereas in permeabilized cells at RT WGA-FITC labeled both surface-bound and internalized NPs, making it possible to quantify NPs that had been internalized and those that were only adsorbed onto the surface [44].

As a transcription factor, OCT4 requires its delivery into the nucleus. Employing a FRET assay to evaluate the nuclear targeting of NPs, we surprisingly found that the presence of an NLS even reduced nuclear targeting of OCT4. Further experiments were therefore conducted with unmodified NPs. Confocal microscopy confirmed the cellular and nuclear entry of intact OCT4-NPs. Prior to microscopy, we treated the permeabilized cells with WGA-Alexafluor 488 to demonstrate that the green fluorescence was due to OCT4-NPs rather than free OCT4. Since WGA preferentially binds to chitosan NPs [44], the detectable yellow fluorescence indicated a co-localization of OCT4 and WGA, suggesting that the signal was due to intracellular rather than free OCT4.

Apart from stabilizing and delivering OCT4 to its site of action, NPs enable the use of high concentrations of reprogramming factors. In the case of OCT4 an encapsulation efficiency of ≈75% was achieved, which allowed cell treatment with a concentration of 100 μg/mL (≈2.6 μM) of OCT4. At this concentration (corresponding to 1 mg/mL chitosan) OCT4 was neither degraded, precipitated nor were the target cells affected.

So far, only two studies employed nanocarriers in protein-induced reprogramming. Cho *et al.* used TiO_2_ nanotubes with electrostatically adsorbed Oct4, Sox2, Klf4 and Nanog for reprogramming of neural stem cells [45]. While the authors observed stem cell-like morphological changes, fully reprogrammed iPSCs were not obtained. It is possible that the electrostatic binding, unlike chitosan encapsulation of proteins, hindered the release of the reprogramming factors. Khan *et al.* obtained iPSC colonies using synthetic surfactants complexed with KLF4, SOX2 and NR5A2 following three rounds of protein transduction [46]. In addition, several other possibilities can enhance the efficiency of iPSC generation. For instance, histone deacetylase inhibitors or activation of toll-like receptor-3 were reported to enhance reprogramming by cell-permeant OSKM fusion proteins by facilitating epigenetic alterations [19, 47].

In contrast to the baculoviral expression yielding high amounts of active OCT4, most studies used bacterially expressed OSKM factors for protein-induced reprogramming attempts. The bacterially overexpressed transcription factors were found in inclusion bodies and had to be denatured and refolded *in vitro*, resulting in a partial loss of activity and reprogramming efficiency [19]. Low efficiencies could generally be attributed to the poor stability and solubility of recombinant factors, their low cellular entry or their poor endosomal release [33–35]. Indeed, a major drawback of PTD fusion proteins is their inefficient release from endosomal vesicles into the cytosol. Interestingly, cell permeabilization with streptolysin O has been reported to enhance protein-induced reprogramming, presumably by avoiding entrapment of OSKM factors in lysosomes [48]. Similarly, sucrose acting as a lysomotrophic agent can increase the reprogramming efficiency of OCT4-TAT [35].

Although cell-permeant PTD-fused OSKM factors enhance the cellular uptake, they do not provide a sustained supply, unless protein treatment is repeated several times. A sustained supply of the OSKM factors is required for reprogramming and a major reason for the high reprogramming efficiency of retroviral expression systems. In this context NP-encapsulated OSKM factors might provide a more sustained supply compared to soluble proteins. Further studies are therefore ongoing to generate NPs for SOX2, c-MYC and KLF4 and to evaluate their utility in protein-based iPSC generation.

## MATERIALS AND METHODS

### Chitosan nanoparticle formulation and characterization

Small and large chitosan NPs were formulated by the inotropic gelation method using low-molecular weight chitosan (50–190 kDa; Sigma-Aldrich, St. Louis, MO) and tripolyphosphate (TPP; Mistral Chemicals, Antrim, UK) [28]. NP average hydrodynamic diameter and zeta potential were determined using a Zetasizer Nano ZS90 device (Malvern Instruments, Herrenberg, Germany). After dilution of the NP suspension with de-ionized water samples were measured in triplicate at 25°C and calculated as means ± SD. For morphological analysis, a drop of the diluted NP suspension was spread on glass slides and subjected to field-emission scanning electron microscopy using a LEO SUPRA 55 microscope (Carl Zeiss, Reutlingen, Germany).

Horseradish peroxidase (HRP; ~150 U/mg; Sigma-Aldrich) was used as a model protein to study protein encapsulation. HRP-loaded chitosan NPs were formulated by dissolving HRP in TPP solution. To exclude that the NP encapsulation of proteins by inotropic gelation did affect protein activity, encapsulation efficiency (EE) was determined based on enzyme activity and protein content. To this end, L-NPs were centrifuged at 14000 g for 30 min, before un-entrapped protein was quantified in the supernatant. S-NPs were centrifuged in Roti^®^-Spin centrifugal filters (molecular weight cut-off 100 kDa; Carl Roth GmbH, Karlsruhe Germany) for 20 min at 4000 g, before the non-entrapped protein was quantified in the flow-through. EE based on total protein content was determined by Coomassie Plus (Pierce Biotechnology, Rockford, IL) following removal of excess chitosan. HRP L-NPs and S-NPs were purified as described above. To 1 mL of L-NP supernatant and S-NP flow-through 20 μL of 20% NaOH were added. Samples were then centrifuged at 14000 g for 5 min, before chitosan-free supernatants were used to quantify un-entrapped protein using Coomassie dye.

Activity-based quantification of HRP was performed as described [49]. Briefly, 1.5 mL of 1.7 mM of hydrogen peroxide in 0.2 M potassium phosphate buffer was mixed with 1.4 mL of 2.5 M 4-aminoantipyrine-phenol (Acros Organics, Geel, Belgium). Increase in absorbance at 510 nm was recorded using a UV–Vis spectrophotometer every 30 sec for 5 min upon addition of 100 μL enzyme-containing test solution (L-NP supernatant or S-NP flow through). Rate of reaction was determined and used to calculate the enzyme concentration from a HRP standard calibration curve.

To determine the ability of NPs to release proteins, HRP-loaded NPs were purified, reconstituted in 1 mL de-ionized water and placed in spectra/por float-A-lyzers (molecular weight cut-off 100 kDa; Spectrum Labs, Breda, The Netherlands). NP-loaded float-A-lyzers were submerged in 6 mL PBS and maintained at 37°C in a shaking water bath. At predetermined time intervals, 200 μL of the samples were removed, replaced with fresh buffer and analyzed for active HRP. All samples were analyzed in triplicate and results are given as means ± SD.

### OCT4 expression and purification

Sf9 insect cells were cultured with EX-Cell 420 media (Sigma-Aldrich) supplemented with 10% fetal calf serum and penicillin/streptomycin. *OCT4* cDNA was cloned into pAcG2T transfer vector which contains GST tag and transferred by homologous recombination to baculoviral bright linear DNA (BD Biosciences, Heidelberg, Germany) containing a *GFP* gene. To obtain recombinant OCT4, Sf9 cells in 1 L suspension cultures were infected with baculovirus at a multiplicity of infection (MOI) of 10. Five days post-infection cells were harvested by centrifugation at 4°C for 10 min at 2000 g and washed twice with 50 mL PBS. As recombinant OCT4 localizes in the nucleus of Sf9 cells, nuclear extraction was conducted as described [50]. Briefly, cell pellets were suspended in hypotonic buffer (10 mM Na-HEPES, pH 7.9, 10 mM KCl, 0.1 mM EDTA, 0.1 mM EGTA, 1 mM DTT and 0.5 mM PMSF) and incubated for 15 min on ice. Cells were then lysed with 25 strokes of a Dounce homogenizer. The cell lysate including nuclei was centrifuged at 4600 rpm at 4°C for 10 min, resuspended in nuclear lysis buffer (20 mM Na-HEPES, pH 7.9, 400 mM KCl, 1 mM EDTA, 1 mM EGTA, 10% glycerol, 1 mM DTT) and incubated at 4°C for 30 min with shaking. Nuclear lysates were filtrated through 5-μm and 0.45-μm filters followed by affinity chromatography on glutathione sepharose to isolate GST-tagged OCT4. All steps were carried out at 4°C. After measurement of protein content, the column eluates were pooled to obtain a final concentration of 1 mg/mL and frozen in 25% glycerol. The purity of the OCT4 preparations was analyzed by silver staining and Western blotting using an OCT4 antibody (Cell Signaling Technology, Frankfurt, Germany).

### Determination of the effect of NPs on OCT4 DNA-binding activity

Electrophoretic mobility shift assays (EMSAs) were performed using Odyssey^®^ Infrared EMSA kit (LI-COR Bioscience, Cambridge, UK). Briefly, recombinant OCT4 was incubated with 1 μl of IRDye^®^ 700 infrared dye-labeled double-stranded oligonucleotide (5′-GCCGAATTTGCATATTTGCATGGCTG-3′), 2 μl of 10 × binding buffer, 2.5 mM DTT, 0.25% Tween-20 and 1 μg of poly(dI-dC) in a total volume of 20 μl for 20 min at RT. Samples were separated on 4% native polyacrylamide gels in 0.5 × Tris-borate-EDTA. The gel was scanned by infrared fluorescence detection on the Odyssey^®^ imaging system. DNA-binding activity of encapsulated OCT4 was determined directly after NP formulation, after storage at 4°C, RT and in cell culture media at 37°C.

### Determination of the effect of NLS density on NP cell surface binding and uptake

Rhodamine isothiocynate (RITC)-coupled BSA was used to explore the effect of an NLS on the cell surface binding and cell uptake of S-NPs. NP encapsulation of BSA-RITC was performed as described [28]. NPs were purified and protein content was determined by fluorometry (FLUOstar Optima, BMG Labtech, Ortenberg, Germany; λex 540 nm; λem 625 nm). As a classical NLS the octapeptide CPKKKRKV (Bio Basic Canada Inc., Ontario, Canada) was used. NLS tagging to NPs was performed via N-succinimidyl 3-[2-pyridyldithio]-propionate (Pierce Biotechnology, Rockford, IL), a SH-NH_2_ cross-linker utilizing the N-terminal SH group in the NLS and cationic amines in chitosan. S-NPs with low (L-NLS; 0.25 NLS/nm^2^), intermediate (I-NLS; 0.5 NLS/nm^2^) and high (H-NLS; 2 NLS/nm^2^) NLS density were synthesized. NLS tagging and characterization of the modified NPs was performed as detailed previously [28].

For NP treatment, human primary fibroblasts were plated in 96-well plates (2 × 10^4^ cells/well) and cultured in RPMI-1640 medium supplemented with 10% fetal calf serum, 2 mM glutamine and 2 mM sodium pyruvate. After 48 h cells were washed with PBS and incubated with purified NP-encapsulated BSA-RITC in serum-containing phenol-red free RPMI-1640. The non-modified and NLS-modified S-NPs were added to the cells at a concentration of 100, 250 and 500 μg/mL. After 24 h, culture supernatants were removed and cells were washed twice with PBS. The amount of cell-associated NPs was calculated by fluorometry from standard curves as described [28, 44].

Based on the preferential affinity of wheat germ agglutinin (WGA) to chitosan, FITC-coupled WGA was exploited to distinguish between cell surface-absorbed and internalized NPs. In non-permeabilized cells at 4°C WGA-FITC is not internalized and only labels extracellular chitosan NPs, whereas in permeabilized cells at RT WGA-FITC can enter the cell and label both surface-bound and internalized chitosan NPs. Therefore, fibroblasts were grown in two 96-well plates and loaded with the non-modified or NLS-modified S-NPs as described above. After 24 h, cells were washed twice with PBS. In the first plate, cells were fixed with 4% paraformaldehyde (PFA) for 15 min, washed with PBS, and permeabilized with 0.1% Triton X-100 for 15 min. Cells were then incubated with 100 μL of 10 μg/mL WGA-FITC (Sigma-Aldrich) for 15 min at RT followed by washings in PBS. In the second plate, cells were incubated on ice for 15 min and then directly treated with WGA-FITC for 15 min on ice. After three washings in PBS, the amount of bound WGA-FITC was determined by fluorometry (λex: 494 nm; λem 518 nm) in both plates as described [44]. Results are expressed as mean ± SD from three experiments performed in triplicate.

### Measurement of NP nuclear localization by FRET spectroscopy

The effect of NLS density on nuclear delivery of S-NPs was assessed in intact human fibroblasts by Förster resonance energy transfer (FRET) fluorometry. To this end, the nuclear DNA dye Hoechst 33258 and FITC from FITC-coupled BSA were used as FRET donor and acceptor, respectively. Formulation and encapsulation of BSA-FITC in unmodified and the different versions of NLS-modified S-NPs was performed as described above and detailed previously [28]. Fibroblasts were seeded at a density of 2 × 10^4^ cells/well in 96-well plates, treated with 250 μg/mL of the NP versions after 48 h and incubated for another 24 h. After aspiration of the culture supernatants cells, cells were stained with Hoechst dye (1.5 μg/mL). Cells were washed 3 times with PBS then analyzed by FRET spectroscopy. Three readings were obtained in the FRET channel (λex 366 nm; −λem 518 nm) either for Hoechst-stained and NP-treated cells, for NP-treated cells or for Hoechst-stained cells. FRET was determined from the increase in FITC emission due to a nuclear colocalization of both dyes. FRET efficiency was calculated as described [28].

### Detection of OCT4 nuclear delivery by confocal laser scanning microscopy

Human fibroblasts were seeded on coverslips in 24-well plates at a density of 5 × 10^5^ cell/well. After 24 h cells were treated with either 50 μg/mL of OCT4 encapsulated in S-NPs or soluble OCT4 and incubated for another 24 h. Following washes in PBS, cells were stained with Hoechst 33258 (1.5 μg/mL) for 5 min, fixed with 4% PFA and permeabilized with 0.1% Triton X-100 in PBS. WGA-Alexafluor 488 (Life Technologies, Darmstadt, Germany) was added to the cells for 15 min at RT. Cells were then washed twice with PBS and incubated in blocking buffer (1% BSA in PBS) for 30 min. OCT4 antibody (1:100) was incubated overnight at 4°C. After three washes in blocking buffer, FITC-conjugated goat anti-rabbit IgG (1:500; Promega, Mannheim, Germany) was applied for 1 h at RT. Coverslips were washed in PBS, mounted in fluorescence-mounting medium (DAKO, Hamburg, Germany) and examined by confocal microscopy (Eclipse Ti, Nikon, Tokyo, Japan).
